# Age- and Sex-Specific In-Hospital Mortality after Myocardial Infarction in Routine Clinical Practice

**DOI:** 10.4061/2010/752765

**Published:** 2010-12-28

**Authors:** Chizobam Ani, Deyu Pan, David Martins, Bruce Ovbiagele

**Affiliations:** ^1^Department of Family Medicine, Charles Drew University of Medicine and Science, Los Angeles, CA 90059-2518, USA; ^2^Department of Internal Medicine, Charles Drew University of Medicine and Science, Los Angeles, CA 90059-2518, USA; ^3^Department of Internal Medicine, University of California at Los Angeles, Los Angeles, CA 90095, USA; ^4^Department of Neurology and Stroke Center, University of California at Los Angeles, Los Angeles, CA 90095, USA

## Abstract

*Background*. Literature regarding the influence of age/sex on mortality trends for acute myocardial infarction (AMI) hospitalizations is limited to hospitals participating in voluntary AMI registries. *Objective*. Evaluate the impact of age and sex on in-hospital AMI mortality using a nationally representative hospital sample. *Methods*. Secondary data analysis using AMI hospitalizations identified from the Nationwide-Inpatient-Sample (NIS). Descriptive and Cox proportional hazards analysis explored mortality trends by age and sex from 1997–2006 while adjusting for the influence of, demographics, co-morbidity, length of hospital stay and hospital characteristics. *Results*. From 1997–2006, in-hospital AMI mortality rates decreased across time in all subgroups (*P* < .001), except for males aged <55 years. The greatest decline was observed in females aged <55 years, compared to similarly aged males, mortality outcomes were poorer in 1997-1998 (RR 1.47, 95% CI  =  1.30–1.66), when compared with 2005-2006 (RR 1.03, 95% CI  =  0.90–1.18), adjusted *P* value for trend demonstrated a statistically significant decline in the relative AMI mortality risk for females when compared with males (<0.001). *Conclusion*. Over the last decade, in-hospital AMI mortality rates declined for every age/sex group except males <55 years. While AMI female-male mortality disparity has narrowed, some room for improvement remains.

## 1. Introduction

Cardiovascular disease is the leading cause of death and disability in the United States, accounting for the underlying cause of death for about 1 in 2.8 deaths in the United States [1, 2]. Fortunately, observations over the last 20 years suggest that death rates following an index AMI are steadily declining [3–7], largely due to effective drugs and revascularization procedures [8, 9]. Recent evidence also indicates that the hitherto reported sex disparity: higher mortality rates after AMI in young females relative to their similarly aged male counterparts may be diminishing [10–13]. 

However, many of the aforementioned studies obtained data from registries comprising hospitals that voluntarily signed up for participation or focused only on a specific age group (e.g., >65 years) and thus may not be broadly representative. Indeed, hospitals participating in these registries tend to differ from nonparticipating hospitals by being larger, more procedure-oriented centers with a major interest in the improvement of quality metrics and processes [14]. 

Using widely representative hospital administrative data, the main objective of this study was to assess trends in age-and sex-specific in-hospital mortality after AMI in the United States. A secondary objective was to examine recent nationwide patterns in the use of common procedures during AMI hospitalization by sex and age.

## 2. Methods

Data were obtained from the Nationwide Inpatient Sample (NIS), developed as part of the Healthcare Cost and Utilization Project (HCUP), a Federal-State-Industry partnership sponsored by the Agency for Healthcare Research and Quality (AHRQ) [15]. NIS is designed to approximate a stratified 20% sample of all non-Federal, short-term, general, and specialty hospitals serving adults in the United States. The sampling strategy selects hospitals within states that have State Inpatient Databases (SID) according to defined strata based on ownership, bed size, teaching status, urban/rural location, and region. All discharges from sampled hospitals for the calendar year are then selected for inclusion into NIS. To allow for national estimate extrapolation, both hospital and discharge weights were provided. Detailed information on the design of the NIS is available at http://www.hcup-us.ahrq.gov. From 1997 to 2006, NIS captured discharge-level information on primary and secondary diagnoses and procedures, discharge vital status, and demographics on discharges per year. Data elements that could directly or indirectly identify individuals were excluded; we thus considered all discharges to be independent. The unit of analysis was the discharge rather than the individual. A unique hospital identifier allows for linkage of discharge data to an NIS data set with hospital characteristics. 

To analyze myocardial infarction hospitalizations, we identified all discharges for which an ICD9-CM code of 410.xx (acute myocardial infarction including STEMI and NSTEMI) was listed as the primary diagnosis. This approach has been utilized by other studies and was taken to specifically focus on patients who presented with acute myocardial infarction and not those patients who had AMI secondary to other conditions like, surgery, hypotension, or other events post hospitalization. Total numbers of myocardial infarctions were obtained by summing across codes. Similarly procedure codes for the ten most common procedures were also identified using the reported ICD9-CM code. We accounted for procedure code changes that occurred in 2005 while extracting the data (single vessel percutaneous transluminal coronary angioplasty [PTCA] or coronary atherectomy with and without a thrombolytic agent; 36.01 and 36.02). This secondary data analysis study was approved by the Charles R Drew University of Medicine and Science IRB.

## 3. Statistical Analyses

We compared trends in-hospital AMI mortality in males and females before and after adjustment for covariates. Analyses were stratified by age group including <55, 55–64, 65–74, 75–84, and >84 years. The following demographic and clinical characteristics were adjusted for: age, race (white, black, other, and unknown), primary payer (Medicare, Medicaid, private, and other), medical comorbidity, and number of procedures performed as ordinal variables. In addition, the following hospital characteristics were also adjusted for region (NE, MW, S, and W), bedsize (small, medium, and large), AMI volume by quartile and location/teaching status (rural, urban nonteaching, and urban teaching). The time variable was grouped into five 2-year intervals from 1997-1998 to 2005-2006. 

The number and severity of comorbid conditions were assessed using the Charlson's co-morbidity index (CCI) [16–18]. We used the modified version of the CCI based on the recent work by Quan et al. [19]. The CCI is a weighted score composed of 17 comorbid conditions including congestive heart failure, myocardial infarction, chronic pulmonary disease, cerebrovascular disease, hemiplegia or paraplegia, dementia, diabetes without complications, diabetes with complication, malignancy, metastatic solid tumor, mild liver disease, moderate or severe liver disease, peptic ulcer disease, peripheral vascular disease, rheumatologic disease, renal disease, and AIDS. 

For the purpose of the multivariate Cox regression analysis, CCI was grouped into 4 categories including a CCI of 1, 2, 3, or 4 or greater. As an additional strategy, the relationship between comorbidities and in-hospital mortality after MI was modeled by including the individual comorbid conditions in the models as separate variables. However, this latter approach gave similar results as when using CCI as a categorical variable and is therefore not reported. In addition, we also adjusted for the following three additional co-morbid conditions that were not components of the CCI: 1.) valvular disease (ICD9 code: 0932, 394, 395, 396, 397, 424, 7463, 7464, 7465, 7466, V422, and V433), hypertension (ICD9 codes 401–405, 6420, 6421, 6422, and 6427), and atrial fibrilation (ICD9 code 4273). 

We computed the descriptive summary statistics for demographic/clinical characteristics by year, sex, and age group. To simplify the presentation, the descriptive results were reported separately in those younger than 65 years and in those aged 65 years or older. Common in-hospital procedure rates and median length of hospital stay (LOS) were also descriptively evaluated using ICD9-CM codes described earlier.

Unadjusted weighted proportions of MI hospitalizations that resulted in death were computed by year, sex, and age group. Results were plotted in order to visually check for any important trends and/or interaction effects in the data. For each sex/age group combination, trends across time were assessed using Cox regression analysis models while adjusting for in-hospital mortality using a composite of the encounter discharge disposition data and reported LOS for incident and censored mortality occurrence. For each age group, the unadjusted relationship between sex and in-hospital mortality across time was summarized using hazard ratios. 

To evaluate the relationship between sex and in-hospital mortality across time while simultaneously adjusting for all of the above covariates, we used the multivariate Cox proportional hazards regression model and accounted for appropriate weighting, clustering, and stratification required for the complex NIS survey design. Adjusted hazard ratio estimates were computed in each age group separately. We used a series of nested models as a way of identifying the variables that accounted for any observed trends in the unadjusted analyses. All data analyses were conducted using SAS (version 9.1; SAS Institute Inc, Cary, NC). Statistical hypotheses were tested using *P* < .05 as the level of statistical significance.

## 4. Results

Tables 1 and 2 attached show the descriptive summary statistics in persons aged less than 65 years and in persons aged 65 years or older, respectively. AMI encounter cases among males and females aged <65 years and ≥65 years remained approximately similar from 1997 to 2006 though trends in other races demonstrated an increase while Caucasian AMI incidence declined slightly. Median, LOS decreased for all age and sex groups from 1997 to 2006 though females aged <65 tended to have a greater in-hospital LOS than males in this same age group. Among all age groups, Medicare and Medicaid coverage for AMI encounters also demonstrated a slight increase when compared from 1997 to 2006. The burden of multiple medical co-morbidity (CCI ≥ 4) also demonstrated an increase for all age groups from 1997 to 2006. Hypertension and diabetes (with or without complications) accounted for the largest associated cardiovascular co-morbidity among AMI encounters for all age groups with lager increases observed for both males and females of all age groups. Among all age and sex groups AMI encounters with no recorded procedures declined from 1997 to 2006. The overall incidence of left heart cardiac catheterization/coronary arteriography/angiocardiography of left heart structures increased for all age groups though the trend demonstrated greater procedure occurrence among males than females.

**Figure 1:**
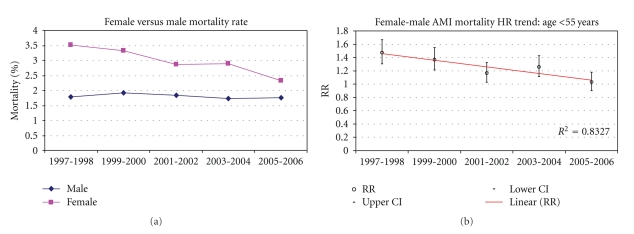
In-hospital AMI mortality rate and relative mortality trend (female versus male) by NIS year for age <55 years.

**Figure 2:**
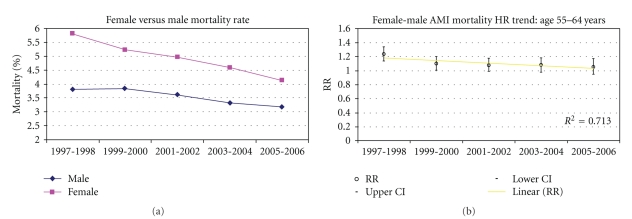
In-hospital AMI mortality rate and relative mortality trend (female versus male) by NIS year for age 55–64 years.

**Figure 3:**
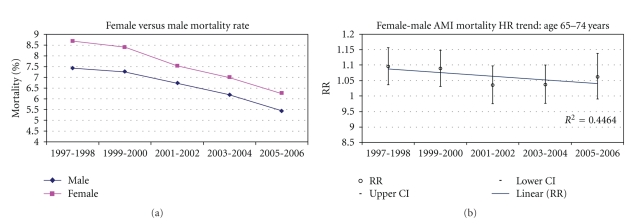
In-hospital AMI mortality rate and relative mortality trend (female versus male) by NIS year for age 65–74 years.

**Figure 4:**
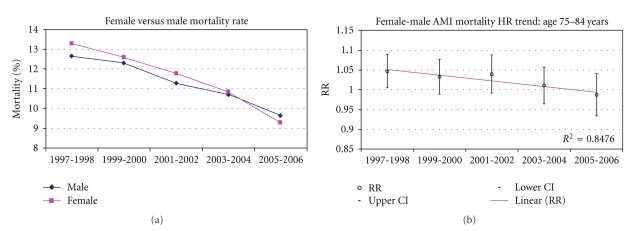
In-hospital AMI mortality rate and relative mortality trend (female versus male) by NIS year for age 75–84 years.

**Figure 5:**
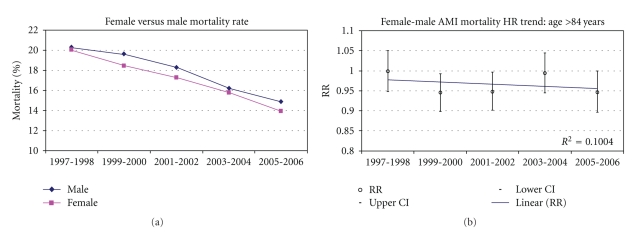
In-hospital AMI mortality rate and relative mortality trend (female versus male) by NIS year for age >84 years.

Tables 3 and 4 demonstrates the crude (unadjusted) age and sex AMI mortality trends from 1997 to 2006. AMI mortality was mostly higher among females when compared with males of younger age groups. A statistically significant decline in AMI mortality was also observed for all age and sex groups except for males aged <55 years for the evaluation period. A linear decline in AMI mortality disparity was observed with increasing age with the most marked difference observed among females age <55 years. In addition, in the younger age groups, mortality rates decreased at a faster rate in females than in males. For example, in the age group 55–64 years, the in-hospital mortality decreased by 30% in 2005-2006 relative to 1997-1998 in females and only by 17% in males. Similarly, in the age group <55 years in-hospital mortality decreased by 35% in 2005-2006 relative to 1997-1998 in females and did not significantly decrease in males. Figures 1, 2, 3, 4 and 5 presents age group-specific trends in male and female AMI mortality for the evaluated year blocks.

Table 5 presents the unadjusted and adjusted female to male relative risk (RR) for in-hospital mortality after acute MI. In MODEL I (unadjusted), the RR difference between females and males was largest in the younger age groups with the difference narrowing with increasing age. In addition, the RR in the younger age groups were larger in the early time points and became smaller at latter time points, indicating that the differences in mortality became less pronounced at latter times (sex by time interaction: *P* < .05 for age groups <65 years). After adjustment for demographic and clinical characteristics (MODEL II), the differences in mortality rates in the younger age groups became less pronounced as evidenced by the smaller RR that were closer to 1 than in the unadjusted analysis. The changes in the RR after the adjustment appear to be uniform across all study years. The sex differences at earlier times, however, remained statistically significant even after the adjustment, implying that the covariates in our model did not fully account for the observed differences between younger males and females at earlier times. In addition, the excess mortality in females relative to males remained higher at earlier time points relative to latter time points, hence the adjustment did not account for the differential sex effect across time. Additional adjustment for hospital characteristics had a negligible effect on the final results (MODEL III). Figures 1–5 present age group-specific AMI relative mortality risk trends in females compared with males (reference) AMI incidence for the evaluated year blocks.

## 5. Discussion

Our results demonstrate that in-hospital mortality after AMI decreased significantly across time in all gender and age subgroups from 1997 to 2006 in the United States. Furthermore, we confirmed results from studies based on data from registries containing voluntarily participating hospitals that a significant decline has occurred in in-hospital mortality among young and middle-aged females in the past decade [10]. These changes in mortality after AMI in young and middle-aged females greatly narrowed the prevailing disparity but by no means eliminated it, indicating that there remains some room for improvement.

Our results also demonstrate that crude in-hospital procedure rates were much less in age group matched females than in their male counterpart, particularly for revascularization procedures though further studies are required to understand these observations in detail. Previous studies have reported that less aggressive therapy including the use of acute reperfusion therapies like percutaneous coronary intervention (PCI) may be contributory to poorer outcomes observed in young and middle-aged females when compared with their male counterparts [20–24]. We found that although crude in-hospital procedure rates were performed with lesser frequency among females, particularly revascularization procedures, the sex differential in crude procedure rates observed in our study did not adequately account for the observed mortality disparity. 

Of note, we did observe that among females aged <65 years the crude multiple co-morbidity rates (>3 CCI) was higher for females compared with males. It is conceivable that additional co-morbidity especially presence/severity of cardiovascular risk factors [25], may influence initiation of aggressive revascularization therapy as well as worsen clinical outcomes in AMI patients. 

The changing co-morbidity and cardio-metabolic disease risk profile in females when compared with males may also contribute to narrowing disparity observations. Risk and severity for adverse cardiovascular is associated with underlying cardiometabolic disease and risk profile [25]. Recent reports indicate a narrowing of risk and co-morbidity burden among females when compared to males. For example, while obesity in the population as a whole has increased in the US population from 1999 to 2008, this increase has been more remarkable among males than females of all ages [26]. Females have been reported to engage in physical activity at a slightly higher rate than males and have also been reported to smoke less cigarettes than males [27]. Some studies also report that females particularly younger females may be more sensitized than they were a decade ago about the risk associated with cardiovascular disease. This factor may inadvertently be associated with facilitated care initiation and improved in-hospital outcomes for females [28]. 

The relative lack of improvement in AMI mortality rates among US males aged <55 years, over the last decade is also worthy of note. As much as the sex disparity in AMI mortality for persons <55 years needs to be bridged, it would be prudent not to overlook young and middle-aged males so that a midlife sex disparity unfavorable to males does not develop in future years.

This study is subject to important limitations including, the use of administrative data that extrapolates AMI occurrence by using ICD-9 coding for incident disease encounters. Coding errors may over or underreport AMI incidence. Observations of AMI incidence without documented procedures recorded may be indicative of false AMI diagnosis in the sample, since it is rare that AMI encounters will exclude diagnostic or therapeutic risk assessment. This observed phenomenon may also indicate secondary referral of patients to centers with more appropriate risk exploration and stratification capacity. Additionally, we could not control for the differential influence of certain risk factors for adverse cardiovascular disease outcomes like obesity, dyslipidemia and smoking status using in-hospital administrative data. In spite of these limitations, we believe this study demonstrates a significant reduction in AMI in-hospital mortality trend from 1997 to 2006, and the most remarkable gain was observed among young females aged <55 years when compared to age-matched males. This study supports the need for continued clinical and public health effort aimed at reducing any residual disparities in outcomes for females when compared with males while improving the overall in-hospital quality of care for AMI.

## Figures and Tables

**Table 1:** Descriptive summary statistics by NIS year and sex: acute MI hospitalizations, age <65 years.

	Year 1997-1998		Year 1999-2000		Year 2001-2002		Year 2003-2004		Year 2005-2006	
	Male	Female	Male	Female	Male	Female	Male	Female	Male	Female
Demographic characteristics	*N* = 418,685	*N* = 150,983	*N* = 415,073	*N* = 154,638	*N* = 431,435	*N* = 164,923	*N* = 417,284	*N* = 161,420	*N* = 399,493	*N* = 155,542

Age^†^, mean	52.8	54.4	52.8	54.1	52.9	54.0	53.0	54.0	53.0	54.0
White	63.2%	59.5%	61.0%	56.4%	54.4%	50.4%	53.0%	49.4%	54.9%	50.3%
Black	6.2%	11.2%	6.1%	11.1%	6.2%	11.2%	6.5%	11.6%	5.9%	10.9%
*Other Race^†^	7.5%	6.5%	8.2%	7.5%	9.6%	8.6%	10.5%	9.9%	10.7%	10.2%
Unknown Race^†^	23.1%	22.8%	24.7%	24.9%	29.9%	29.8%	30.0%	29.1%	28.5%	28.6%

Primary payer										

Medicare	9.9%	12.5%	10.9%	14.3%	11.3%	15.0%	11.6%	16.3%	11.9%	17.1%
Medicaid	6.8%	14.0%	7.2%	14.5%	7.6%	14.6%	8.4%	15.7%	8.6%	15.4%
Private	69.3%	60.1%	68.0%	58.1%	66.5%	57.1%	63.4%	52.9%	61.3%	51.1%
**Other	14.0%	13.4%	13.8%	13.0%	14.6%	13.4%	16.7%	15.1%	18.3%	16.4%

Charlson's comorbidity index										

1	58.7%	43.0%	57.0%	40.6%	55.9%	40.4%	53.4%	37.6%	51.9%	36.3%
2	26.0%	30.5%	26.4%	30.8%	26.6%	30.5%	27.4%	30.6%	27.7%	30.7%
3	9.2%	14.8%	9.6%	15.8%	9.8%	15.4%	10.8%	16.5%	11.0%	16.5%
≥4	6.1%	11.7%	7.0%	12.7%	7.7%	13.7%	8.3%	15.3%	9.4%	16.6%

Cardiovascular Comorbid condition										

Atrial fibrillation/flutter	6.3%	5.8%	6.1%	5.4%	6.5%	5.6%	6.7%	6.0%	6.9%	6.1%
Cerebrovascular disease	2.2%	3.5%	2.3%	3.5%	2.2%	3.6%	2.3%	3.6%	2.3%	3.6%
Chronic pulmonary disease	11.5%	16.2%	12.1%	18.1%	12.6%	19.1%	13.6%	21.1%	14.2%	22.3%
Congestive heart failure	15.4%	22.5%	15.1%	22.3%	15.1%	21.8%	16.3%	23.8%	16.2%	23.5%
Diabetes with complication	2.7%	5.9%	2.9%	6.2%	3.1%	6.1%	3.1%	6.4%	3.0%	5.8%
Diabetes without complication	17.8%	27.1%	19.6%	28.2%	20.9%	28.5%	22.0%	30.1%	22.8%	31.2%
Hypertension	40.8%	48.4%	43.6%	51.9%	47.6%	55.3%	52.0%	58.5%	56.2%	61.6%
Peripheral vascular disease	3.6%	4.7%	4.0%	5.2%	4.0%	5.7%	4.6%	6.0%	4.9%	6.3%
Renal disease	2.5%	4.5%	3.1%	5.7%	3.6%	6.2%	3.9%	6.9%	5.7%	8.7%
Valvular disease	5.9%	9.6%	5.0%	8.3%	5.1%	8.4%	5.4%	8.4%	5.8%	9.4%

Number of procedures performed										

0-1										
2-3	26.1%	27.4%	21.8%	24.4%	18.6%	22.1%	15.6%	20.3%	12.5%	17.9%
4-5	27.5%	24.7%	25.9%	24.3%	26.3%	24.4%	26.2%	24.2%	17.6%	18.2%
≥6	18.8%	17.6%	26.3%	22.5%	33.3%	28.3%	40.4%	33.6%	55.8%	45.2%

Type of procedure performed										

3722/8856/8853—Left Heart cardiac catheterization/coronary arteriography/angiocardiography of left heart structures	60.82%	56.94%	63.39%	58.63%	68.20%	63.57%	73.42%	67.29%	77.08%	71.52%
3601 and 3602—single-vessel PTCA or coronary atherectomy with or without mention of thrombolytic agent	32.53%	28.16%	35.08%	29.56%	40.80%	33.26%	44.79%	36.10%	42.20%	33.76%
3606/3607—insertion of nondrug/ drug-eluting coronary artery stent(s)	25.13%	20.71%	34.81%	28.28%	42.30%	33.46%	48.55%	37.95%	54.45%	42.50%
9920—injection or infusion of platelet inhibitor	0.6%	0.4%	14.6%	11.5%	23.7%	18.9%	27.6%	21.5%	25.6%	20.1%
3961—extracorporeal circulation auxiliary to open heart surgery	10.8%	8.7%	9.9%	7.7%	9.5%	7.1%	9.3%	6.7%	8.2%	5.8%
3615—single internal mammary-coronary Artery bypass	9.6%	7.3%	9.7%	7.2%	10.1%	7.3%	9.9%	7.0%	9.7%	6.8%
8872^†^—diagnostic ultrasound of heart (Echo, TEE)	6.0%	6.4%	4.7%	5.1%	4.2%	4.6%	5.2%	5.7%	5.0%	5.0%
No procedure performed	18.4%	20.8%	18.1%	20.7%	15.8%	18.8%	13.1%	16.1%	10.5%	13.9%
LOS, median days	3.2	3.5	2.8	3.1	2.6	2.9	2.6	2.8	2.4	2.7

*includes Hispanic, Asian Pacific Islander, Native American, and other.

**includes no pay, self pay, and other.

^†^indicates *P* > .05 (not significant) as follows; (a) Age for all year groups, (b) Other race: Year 2003-2004 and Year 2005-2006, (c) Unknown race for all year groups, and (d) Procedure 8872 for all year groups.

**Table 2:** Descriptive summary statistics by NIS year and sex: acute MI hospitalizations, age ≥65 years.

	Year 1997-1998		Year 1999-2000		Year 2001-2002		Year 2003-2004		Year 2005-2006	
	Male	Female	Male	Female	Male	Female	Male	Female	Male	Female
Demographic characteristics	*N* = 471,238	*N* = 436,874	*N* = 469,844	*N* = 451,786	*N* = 473,381	*N* = 465,064	*N* = 436,054	*N* = 428,289	*N* = 396,588	*N* = 382,075

Age^†^, mean	75.4	78.2	75.9	78.8	76.3	79.2	76.5	79.4	76.6	79.7
White	70.6%	68.6%	68.3%	66.1%	62.3%	60.5%	60.3%	58.8%	61.8%	60.2%
Black	3.9%	5.4%	4.0%	5.6%	3.9%	5.4%	4.4%	6.2%	3.8%	5.6%
*Other race	5.8%	5.4%	6.5%	6.1%	7.3%	6.8%	8.5%	8.1%	8.5%	8.0%
Unknown race^†^	19.6%	20.6%	21.2%	22.2%	26.6%	27.3%	26.7%	26.9%	25.9%	26.2%

Primary payer										

Medicare	84.1%	88.1%	85.6%	89.7%	86.8%	90.6%	88.4%	91.7%	88.0%	91.9%
Medicaid	1.0%	1.4%	1.2%	1.6%	1.2%	1.5%	1.1%	1.6%	1.0%	1.4%
Private	13.4%	9.5%	11.6%	7.6%	10.4%	6.9%	8.9%	5.7%	9.2%	5.7%
**Other	1.6%	1.0%	1.7%	1.2%	1.5%	1.0%	1.6%	1.1%	1.8%	1.0%

Charlson's comorbidity index										

1	30.8%	26.8%	28.9%	25.1%	28.2%	24.3%	26.1%	22.0%	24.4%	20.8%
2	31.3%	34.2%	31.1%	33.6%	29.9%	32.7%	29.7%	32.2%	27.0%	29.8%
3	20.1%	22.5%	20.8%	23.4%	21.1%	23.5%	21.8%	24.5%	20.4%	23.3%
≥4	17.8%	16.5%	19.2%	17.9%	20.8%	19.5%	22.4%	21.2%	28.2%	26.0%

Cardiovascular Comorbid condition										

Atrial atrial fibrillation /flutter^†^	22.2%	21.6%	22.7%	22.0%	23.2%	23.0%	23.8%	23.2%	24.4%	24.6%
Cerebrovascular disease	7.4%	8.2%	7.4%	8.4%	7.0%	8.1%	7.0%	8.2%	6.9%	8.2%
Chronic pulmonary disease	21.4%	17.6%	23.3%	19.5%	24.3%	21.6%	25.0%	23.4%	26.1%	25.6%
Congestive heart failure	38.9%	46.3%	39.2%	46.6%	39.0%	46.1%	40.7%	48.1%	40.6%	48.0%
Diabetes with complication^†^	3.4%	3.8%	3.7%	4.0%	3.9%	4.0%	4.2%	4.0%	4.1%	4.0%
Diabetes without complication	23.2%	25.8%	24.5%	26.8%	25.6%	26.9%	26.6%	27.8%	27.3%	27.8%
Hypertension	45.4%	53.9%	49.7%	57.5%	54.3%	61.4%	59.0%	65.1%	63.5%	69.0%
Peripheral vascular disease	9.0%	7.2%	9.6%	7.7%	10.1%	8.3%	10.8%	9.0%	11.6%	9.6%
Renal disease	6.7%	5.6%	7.7%	6.5%	9.2%	7.6%	10.5%	8.7%	17.8%	14.2%
Valvular disease	15.5%	18.8%	13.3%	17.2%	14.1%	17.7%	14.8%	18.3%	16.8%	20.5%

Number of procedures performed										

0-1	41.6%	49.0%	40.9%	49.6%	38.0%	47.2%	35.4%	44.8%	32.4%	42.5%
2-3	23.0%	22.0%	20.8%	19.9%	18.4%	18.3%	17.2%	17.8%	15.1%	15.9%
4-5	18.0%	15.4%	17.9%	14.9%	18.0%	15.0%	18.2%	15.5%	14.2%	12.6%
≥6	17.5%	13.7%	20.5%	15.7%	25.6%	19.5%	29.3%	22.0%	38.3%	28.9%

Type of procedure performed										

3722/8856/8853—left heart cardiac Catheterization/Coronary Arteriography/angiocardiography of left heart structures	42.46%	34.48%	43.52%	34.84%	47.73%	38.52%	50.35%	40.62%	53.86%	43.90%
3601 and 3602—single vessel PTCA or coronary Atherectomy with or without mention of thrombolytic agent	16.88%	14.38%	18.03%	14.81%	21.79%	17.59%	23.97%	19.15%	23.44%	18.71%
3606/3607—insertion of nondrug/Drug-eluting coronary artery stent(s)	12.76%	10.34%	17.80%	14.39%	22.47%	18.05%	26.06%	20.67%	30.52%	24.12%
9920^†^—injection or infusion of platelet inhibitor	0.3%	0.2%	7.4%	5.8%	13.3%	10.0%	14.7%	11.3%	14.3%	10.7%
3961—extracorporeal circulation auxiliary to open heart surgery	10.3%	6.9%	9.2%	5.5%	8.8%	5.4%	8.6%	4.9%	7.8%	4.4%
3615—single internal mammary-coronary artery bypass	8.4%	4.8%	8.5%	4.7%	8.9%	5.0%	8.6%	4.6%	9.1%	4.8%
8872^†^—diagnostic ultrasound of heart (Echo, TEE)	6.8%	7.5%	5.8%	6.0%	5.2%	5.2%	6.0%	6.1%	6.1%	6.0%
No procedure performed	31.1%	37.5%	31.1%	38.5%	29.4%	37.1%	26.8%	34.4%	24.4%	32.5%
LOS, median days	4.3	4.6	3.9	4.3	3.7	4.1	3.7	4.0	3.5	3.8

*includes Hispanic, Asian Pacific Islander, Native American, and other.

**includes no pay, self-pay, and other.

^†^indicates *P* > .05 (not significant) as follows: (a) Age for all year groups, (b) Unknown race: Year 2001-2002, Year 2003-2004, and Year 2005-2006, (c) Procedure 9920 for Year 1997-1998, (d) Procedure 8872 for all year groups, (e) Diabetes with complication Year 2001-2002 and Year 2005-2006 (f) Atrial fib Year 2001-2002 and Year 2005-2006.

**Table 3:** In-hospital AMI mortality rate by NIS year, sex, and age group.

Age group	NIS year	% Mortality (male)	SE	% Mortality (female)	SE
<55 yrs	1997-1998	1.78%	0.07%	3.50%	0.17%
	1999-2000	1.91%	0.07%	3.33%	0.15%
	2001-2002	1.83%	0.07%	2.87%	0.14%
	2003-2004	1.74%	0.08%	2.89%	0.14%
	2005-2006	1.76%	0.07%	2.32%	0.12%

55–64 yrs	1997-1998	3.81%	0.11%	5.80%	0.19%
	1999-2000	3.84%	0.11%	5.23%	0.18%
	2001-2002	3.61%	0.11%	4.97%	0.17%
	2003-2004	3.32%	0.11%	4.59%	0.18%
	2005-2006	3.17%	0.10%	4.14%	0.17%

65–74 yrs	1997-1998	7.41%	0.14%	8.70%	0.18%
	1999-2000	7.25%	0.14%	8.40%	0.17%
	2001-2002	6.73%	0.14%	7.53%	0.19%
	2003-2004	6.19%	0.15%	7.00%	0.18%
	2005-2006	5.42%	0.15%	6.25%	0.17%

75–84 yrs	1997-1998	12.64%	0.19%	13.29%	0.19%
	1999-2000	12.31%	0.21%	12.58%	0.20%
	2001-2002	11.28%	0.20%	11.76%	0.19%
	2003-2004	10.72%	0.19%	10.83%	0.19%
	2005-2006	9.64%	0.20%	9.28%	0.19%

>84 yrs	1997-1998	20.27%	0.40%	20.03%	0.32%
	1999-2000	19.59%	0.38%	18.45%	0.27%
	2001-2002	18.30%	0.36%	17.29%	0.27%
	2003-2004	16.24%	0.33%	15.77%	0.26%
	2005-2006	14.86%	0.32%	13.93%	0.24%

**Table 4:** In-hospital AMI mortality change across time (1997–2006).

Sex	Age group	HR change (2005-2006 versus 1997-1998)	95% CI		*P* value for trend
			Lower	Upper	
Males	<55 yrs	0.99	0.88	1.11	0.3690
	55–64 yrs	0.83	0.76	0.90	<.0001
	65–74 yrs	0.72	0.67	0.77	<.0001
	75–84 yrs	0.74	0.70	0.78	<.0001
	>84 yrs	0.69	0.64	0.74	<.0001

Females	<55 yrs	0.65	0.57	0.75	<.0001
	55–64 yrs	0.70	0.63	0.78	<.0001
	65–74 yrs	0.70	0.65	0.75	<.0001
	75–84 yrs	0.67	0.63	0.70	<.0001
	>84 yrs	0.65	0.61	0.68	<.0001

**Table 5:** Univariate (unadjusted) and multivariable (adjusted) Cox regression analysis models for in-hospital AMI mortality NIS year and age group: female versus male (male = reference).

	Year 1997-1998		Year 1999-2000		Year 2001-2002		Year 2003-2004		Year 2005-2006		*P* value for sex-time trend	*P* value for sex-time-age trend
Age Group	RR	95% CI	RR	95% CI	RR	95% CI	RR	95% CI	RR	95% CI		
MODEL I: Unadjusted												

<55 yrs	**1.81**	**1.62**–**2.03**	**1.60**	**1.43**–**1.79**	**1.43**	**1.28**–**1.60**	**1.52**	**1.35**–**1.71**	**1.23**	**1.08**–**1.39**	<.001	*P* < .001
55–64 yrs	**1.38**	**1.27**–**1.49**	**1.25**	**1.15**–**1.35**	**1.24**	**1.14**–**1.35**	**1.26**	**1.15**–**1.37**	**1.21**	**1.09**–**1.33**	.131	
65–74 yrs	**1.11**	**1.06**–**1.17**	**1.11**	**1.05**–**1.17**	**1.06**	**1.00**–**1.13**	**1.08**	**1.02**–**1.14**	**1.09**	**1.02**–**1.17**	.506	
75–84 yrs	1.03	0.99–1.07	1.02	0.98–1.06	1.03	0.99–1.08	1.00	0.96–1.05	0.98	0.93–1.04	.188	
>84 yrs	0.98	0.94–1.04	0.93	0.88–0.98	0.95	0.90–1.00	1.01	0.96–1.06	0.97	0.92–1.02	.760	

MODEL II: Adjusted for demographic and clinical characteristics												

<55 yrs	**1.47**	**1.31**–**1.67**	**1.37**	**1.21**–**1.54**	**1.16**	**1.03**–**1.32**	**1.26**	**1.11**–**1.43**	**1.03**	**0.90**–**1.18**	<.001	*P* < .001
55–64 yrs	**1.23**	**1.13**–**1.34**	**1.10**	**1.01**–**1.21**	**1.08**	**1.00**–**1.18**	**1.08**	**0.98**–**1.18**	**1.06**	**0.95**–**1.17**	.010	
65–74 yrs	**1.09**	**1.03-1.15**	**1.09**	**1.03**–**1.15**	**1.04**	**0.98**–**1.10**	**1.03**	**0.98**–**1.10**	**1.06**	**0.99**–**1.14**	.294	
75–84 yrs	1.05	1.00–1.09	1.03	0.99–1.08	1.04	0.99–1.09	1.01	0.96–1.05	0.99	0.94–1.04	.047	
>84 yrs	1.00	0.95–1.05	0.94	0.90–0.99	0.95	0.90–1.00	0.99	0.95–1.04	0.95	0.90–1.00	.620	

MODEL III: Adjusted for demographic, clinical characteristics and hospital factors												

<55 yrs	**1.47**	**1.30**–**1.66**	**1.37**	**1.21**–**1.55**	**1.16**	**1.03**–**1.32**	**1.26**	**1.11**–**1.43**	**1.03**	**0.90**–**1.18**	<.001	*P* < .001
55–64 yrs	**1.24**	**1.14**–**1.34**	**1.10**	**1.01**–**1.21**	**1.08**	**0.99**–**1.18**	**1.08**	**0.98**–**1.19**	**1.06**	**0.95**–**1.17**	.010	
65–74 yrs	**1.10**	**1.04**–**1.16**	**1.09**	**1.03**–**1.15**	**1.04**	**0.98**–**1.10**	**1.04**	**0.98**–**1.10**	**1.06**	**0.99**–**1.14**	.248	
75–84 yrs	**1.05**	**1.01**–**1.09**	**1.03**	0.99–1.08	1.04	0.99–1.09	1.01	0.96–1.06	0.99	0.93–1.04	.043	
>84 yrs	**1.00**	**0.95**–**1.05**	**0.95**	0.90–0.99	0.95	0.90–1.00	0.99	0.95–1.04	0.95	0.90–1.00	.533	
